# Supplementary material for: A Global Screen for Assembly State Changes of the Mitotic Proteome by SEC-SWATH-MS
Source: Cell Syst. 2020 Feb 26;10(2):133–155.e6. doi: 10.1016/j.cels.2020.01.001 (PMC7042714; doi:10.1016/j.cels.2020.01.001)

Q9BUR5 | MIC26_HUMAN | APOO FAM121B MIC23 MIC26 My025 UNQ1866/PRO4302
Monomer MW [kDa]: 22.285 Monomer expected elution fraction: 53

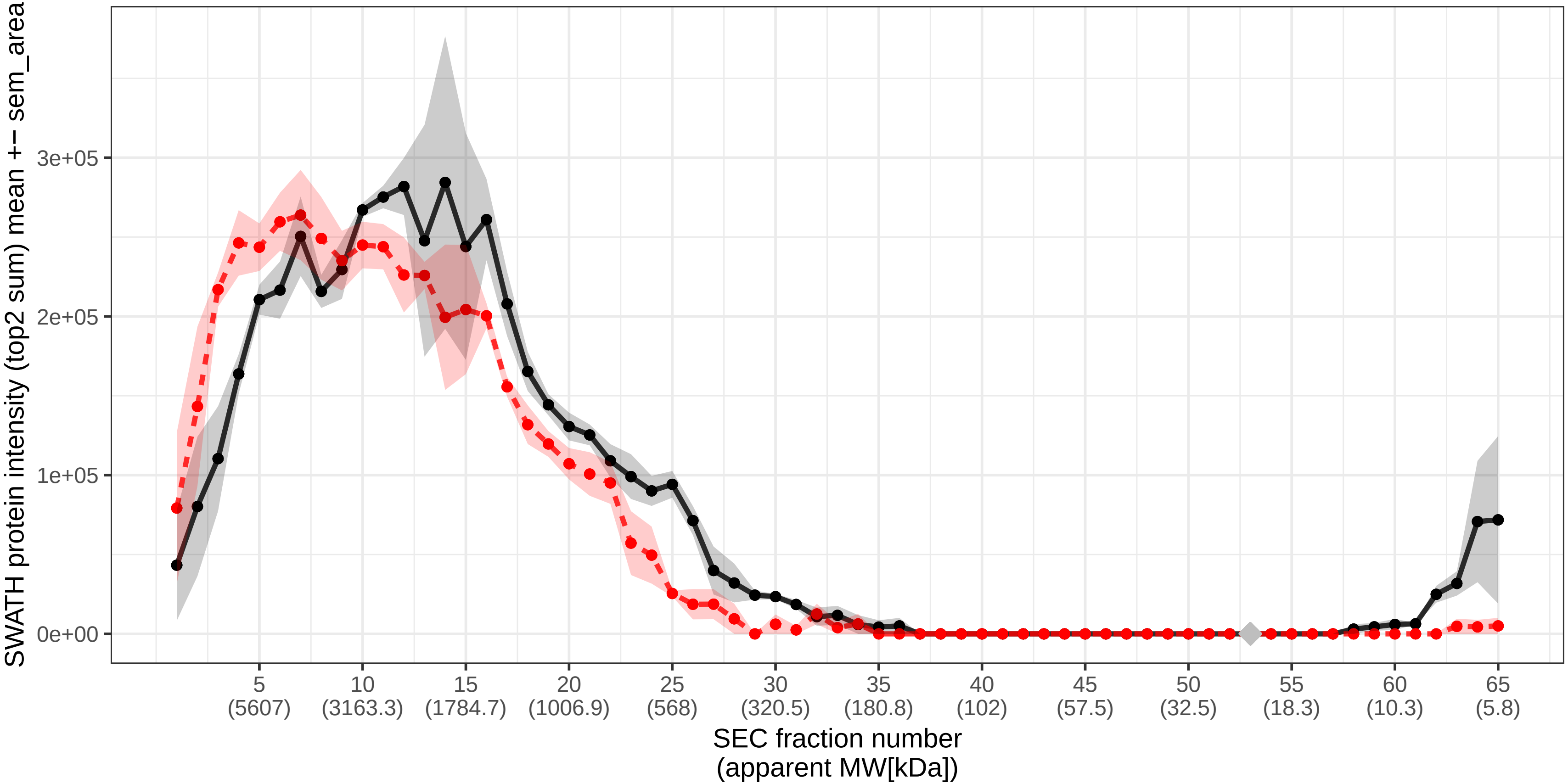

Supplement: Data S1. SEC-SWATH-MS Protein Chromatograms, Related to Figure 1 [file mmc6.zip › SECchrom_Q9BUR5_MIC26_HUMAN_APOO_FAM121B_MIC23_MIC26_My025_.pdf]
